# Seasonality in submesoscale turbulence

**DOI:** 10.1038/ncomms7862

**Published:** 2015-04-21

**Authors:** Jörn Callies, Raffaele Ferrari, Jody M. Klymak, Jonathan Gula

**Affiliations:** 1 MIT/WHOI Joint Program in Oceanography, Massachusetts Institute of Technology, Building 54-1615, 77 Massachusetts Avenue, Cambridge/Woods Hole, Massachusetts 02139, USA; 2 Massachusetts Institute of Technology, Cambridge, Massachusetts 02139, USA; 3 University of Victoria, Victoria, British Columbia V8W 3P6, Canada; 4 University of California, Los Angeles, California 90095, USA

## Abstract

Although the strongest ocean surface currents occur at horizontal scales of order 100 km, recent numerical simulations suggest that flows smaller than these mesoscale eddies can achieve important vertical transports in the upper ocean. These submesoscale flows, 1–100 km in horizontal extent, take heat and atmospheric gases down into the interior ocean, accelerating air–sea fluxes, and bring deep nutrients up into the sunlit surface layer, fueling primary production. Here we present observational evidence that submesoscale flows undergo a seasonal cycle in the surface mixed layer: they are much stronger in winter than in summer. Submesoscale flows are energized by baroclinic instabilities that develop around geostrophic eddies in the deep winter mixed layer at a horizontal scale of order 1–10 km. Flows larger than this instability scale are energized by turbulent scale interactions. Enhanced submesoscale activity in the winter mixed layer is expected to achieve efficient exchanges with the permanent thermocline below.

## Abstract (web summary)

Recent numerical simulations suggest that the fronts that develop along the rims of ocean eddies are stronger in winter than in summer. Here, the authors present observational confirmation, which informs how these frontal flows are formed.

## Graphical abstract

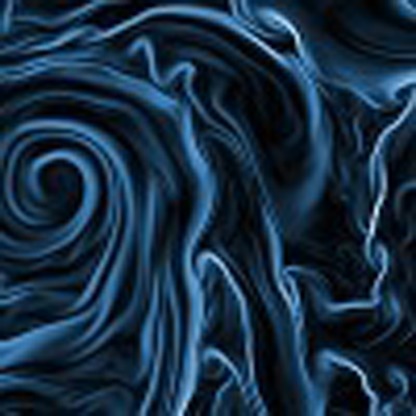

The ocean supports motions on a wide range of scales. The most energetic flows are eddies with horizontal scales of a few hundred kilometres, which are the oceanic analogues of the atmospheric cyclones and anticyclones. These mesoscale eddies are dynamically well understood and are routinely observed with satellite altimeters^1^. Classic theories predict that the kinetic energy in the mesoscale eddy field should decay very rapidly at smaller scales^2^ and be associated with weak vertical velocities. Recent numerical modelling studies suggest, however, that there is a very dynamic near-surface eddy field on scales between 1 and 100 km, which drives vertical velocities one or two orders of magnitude larger than those of order 1 m per day associated with the divergence of mesoscale flows^3^,^4^. These submesoscale flows consist of narrow horizontal currents associated with strong horizontal gradients in buoyancy.

High-resolution numerical simulations suggest that submesoscale flows are much stronger in winter than in summer^5^,^6^,^7^. This is illustrated here with snapshots from a simulation of the Gulf Stream region with a horizontal resolution of 750 m. More detail on the simulation is given in the Methods section. The snapshots of surface buoyancy gradients show that fronts are strong in winter and much less pronounced in summer, except for the Gulf Stream front that persists throughout the year (Fig. 1). This seasonality provides important clues to the dynamics of submesoscale turbulence.

One mechanism that has been proposed to energize submesoscale flows is mesoscale-driven surface frontogenesis^3^: the presence of the ocean surface allows strain fields at the edges of mesoscale eddies to sharpen surface buoyancy gradients^8^,^9^. Unlike in the ocean interior, upwelling of dense water and downwelling of light water cannot counteract the increase of lateral buoyancy gradients at the surface, where the vertical transport of waters must vanish. The resulting surface fronts have been shown to break up in secondary roll-up instabilities, which drive a nonlinear cascade of surface buoyancy variance and energize the entire range of scales below the mesoscale^10^. Surface quasi-geostrophic theory has been used to qualitatively illustrate these dynamics and predicts surface kinetic and potential energy spectra to scale like *k*^−5/3^ (ref. 11), where *k* is the horizontal wavenumber. Surface quasi-geostrophic flows are predicted to be confined to a shallow surface layer; they decay exponentially away from the surface, with smaller-scale motions decaying more rapidly than larger-scale ones. Energy spectra are therefore expected to fall off with wavenumber more rapidly away from the surface. Surface quasi-geostrophic dynamics are modified by ageostrophic effects that accelerate frontogenesis^12^, lead to true frontal collapse and thereby produce *k*^−2^ energy spectra^13^.

Another mechanism that has been proposed to energize submesoscale flows in the mixed layer is baroclinic instability^14^,^15^. This instability allows perturbations to extract energy from the lateral buoyancy gradients generated by mesoscale stirring or by spatial variations in atmospheric forcing. Eady edge waves can propagate both along the surface and along the sharp increase in stratification at the base of the mixed layer^10^,^16^,^17^,^18^,^19^. Edge waves with scales close to the mixed-layer deformation radius, *Nh*/*f*, propagate at the same speed along the surface and the base of the mixed layer, and can therefore constructively interfere (*N* is the density stratification of the mixed layer, *h* is its depth, *f* is the Coriolis parameter). This interference results in an amplification of these disturbances on a time scale *N*/*f*Λ (ref. 16) (Λ is the geostrophic shear in the mixed layer), which causes water from the denser side of the lateral buoyancy gradient to slide under the more buoyant waters. The most unstable mode in the Eady model, which assumes that *N* and Λ are constant through the mixed layer and that the base of the mixed layer is rigid, has a wavelength of 4*Nh*/*f* and a growth rate of 0.3 *f*Λ/*N*^16^,^20^. In deep mixed layers of a few hundred metres and for typical values of *f*∼10^−4^ s^−1^, Λ∼10^−4^ s^−1^ and *N*∼10^−3^ s^−1^, disturbances therefore grow on horizontal scales of order 1–10 km and time scales of order 1 day (ref. 15). Once these disturbances have grown to finite amplitude, turbulent scale interactions exchange kinetic energy between scales. If the flow is sufficiently constrained by the Earth's rotation, kinetic energy is preferentially transferred to larger scales, filling in the spectrum between the mixed-layer deformation radius and the mesoscale^7^,^15^,^21^,^22^. A major prediction of this scenario, which distinguishes it from mesoscale-driven surface frontogenesis, is that the full depth of the mixed layer is energized and that the energy decays rapidly below the base of the mixed layer.

The energization of the submesoscale by such mixed-layer instabilities depends crucially on the mixed-layer depth. Mixed-layer baroclinic instabilities are powered by the potential energy stored in lateral buoyancy gradients. The deeper the mixed layer, for a given lateral buoyancy gradient, the more potential energy is released by the slumping isopycnals and the more energetic the resulting submesoscale flows. Given that lateral buoyancy gradients are fairly constant throughout the year, the kinetic energy generated by the instabilities is thus expected to peak in winter, when mixed layers are deepest. In summer, when mixed layers are shallow, the instabilities can easily be damped out. Although the growth rate of mixed-layer instabilities is independent of the mixed-layer depth *h*, the damping of mixed layer motions by atmospheric forcing is faster in shallower mixed layers^23^. In addition, the mixed-layer deformation radius scales with *h*, so the instability scale is smaller when the mixed layer is shallow. As smaller-scale flows can more easily escape the influence of rotation, kinetic energy created in shallow mixed layers may cascade to smaller rather than larger scales, so that the instability fails to energize the range between the mixed-layer deformation radius and the mesoscale^24^,^25^.

The instability mechanism therefore implies a seasonal cycle in submesoscale turbulence. In winter, when the mixed layer is deep, instabilities grow on a time scale of about 1 day and at a horizontal scale between 1 and 10 km, subsequently energizing the entire submesoscale range through turbulent scale interactions. In summer, the instability is either damped out or fails to energize the submesoscale range because of the lack of a strong inverse cascade, when the mixed layer deformation radius is very small. The mesoscale-driven surface frontogenesis mechanism, on the other hand, only depends on the presence of a mesoscale eddy field, which does not undergo a strong seasonal cycle. The strong seasonality at the submesoscale visible in numerical simulations (Fig. 1) supports the instability mechanism.

Observational confirmation of a seasonal cycle in submesoscale turbulence, however, is so far lacking. Numerical models used for studying submesoscale dynamics typically do not explicitly resolve small-scale processes in the mixed layer. In summer, for example, when the mixed layer is shallow, the models do not resolve the instability scale of the baroclinic instability in the mixed layer, which can be as small as 10–100 m. High-resolution satellite observations show a seasonal cycle in sea surface temperature gradients in the Gulf Stream region^26^, but it remains unclear whether the temperature variations are reflected in density or compensated by salinity variations^27^, whether the temperature signal is accompanied by along-front flows, whether these flows are in geostrophic balance and what the subsurface structure of these flows is.

*In-situ* observations of velocity and buoyancy in the upper ocean are used in this study to conclusively show that submesoscale flows undergo a strong seasonal cycle on scales of 1–100 km. An enhancement of the observed submesoscale flows throughout the winter mixed layer and the flows' leading-order horizontal non-divergence are argued to be further evidence for the instability mechanism.

## Results

### Data

We present data from the western subtropical North Atlantic, where the mixed layer is known to undergo a strong seasonal cycle^28^. The data were collected as part of two separate observational programmes: the Oleander project along a transect between Elisabeth, New Jersey, and Hamilton, Bermuda, occupied weekly in 2005–2013, and the Lateral Mixing Experiment (LatMix) along several straight transects off Cape Hatteras in June 2011 (summer) and just south of the Gulf Stream extension in March 2012 (winter)^29^ (Fig. 2). We consider only the Oleander data southwest of the Gulf Stream extension and separate the transects into winter ones (January through March, when the mixed layers are deepest^28^) and summer ones (June through August). In the Oleander region, mixed layers are on average 150–200 m deep in winter and shoal to order 10 m in summer^28^. In the LatMix winter experiment, the mixed layer was about *h*=220 m deep and had an average stratification of *N*=2 × 10^*−*3^ s^*−*1^, giving an Eady instability scale 4*Nh*/*f*=20 km. In the LatMix summer experiment, the mixed layer was no deeper than *h*=5 m and although there were no shallow measurements of stratification, the Eady instability scale was most likely no larger than 100 m.

Power spectra are computed at constant depths for the longitudinal (along-track) velocities *u*, the transverse (across-track) velocities *v* (both obtained with acoustic Doppler current profilers, ADCPs) and the buoyancy *b* (obtained from towed conductivity–temperature–depth sensors, available only for the LatMix data):


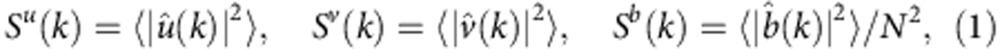


where *k* is the along-track wavenumber, the caret denotes a Fourier transform and 〈·〉 denotes the average over transects. More detail on the observations and the computation of these spectra is provided in the Methods section. Departing from recent literature, we do not focus solely on spectral slopes, because they do not uniquely determine the dynamics: different dynamics can produce the same spectral slopes. We instead use power spectra primarily to diagnose submesoscale energy levels at different times and depths. Together with the relationships between the spectra of *u*, *v* and *b*, this allows a more nuanced assessment of submesoscale dynamics.

### Seasonality

The near-surface spectra of kinetic and potential energy,


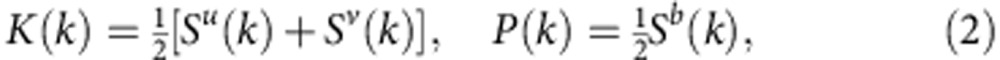


at 50 m depth for Oleander and 20 m depth for LatMix are shown in Fig. 3. The kinetic energy spectra from Oleander and LatMix have similar shapes, suggesting that the dynamics are similar across the different geographical locations. All spectra exhibit marked differences between summer and winter. While the energy levels at the mesoscale (scales larger than 100 km) remain fairly constant across seasons, the submesoscale energy levels (scales of order 10 km) undergo a seasonal transition: they are significantly higher in winter than in summer (Fig. 3). This seasonal difference in submesoscale energy levels is reflected in how rapidly the energy falls off with wavenumber in the submesoscale range. In winter, the spectra are relatively flat and approximately follow a *k*^−2^ power law (Fig. 3a,b). In summer, the spectra in the range 20–100 km fall off more rapidly and approximately follow a *k*^−3^ power law (Fig. 3c,d); at scales smaller than 20 km, the spectra flatten out and roughly match the Garrett–Munk (GM) empirical model spectrum of internal waves^30^. Potential and kinetic energy spectra are approximately equal in the LatMix winter experiment (Fig. 3b). In the LatMix summer experiment, the kinetic energy spectrum is considerably larger than the potential energy spectrum at scales larger than 20 km (Fig. 3d). At smaller scales, they are roughly equal and consistent with the GM spectrum for internal waves.

**Figure 1:**
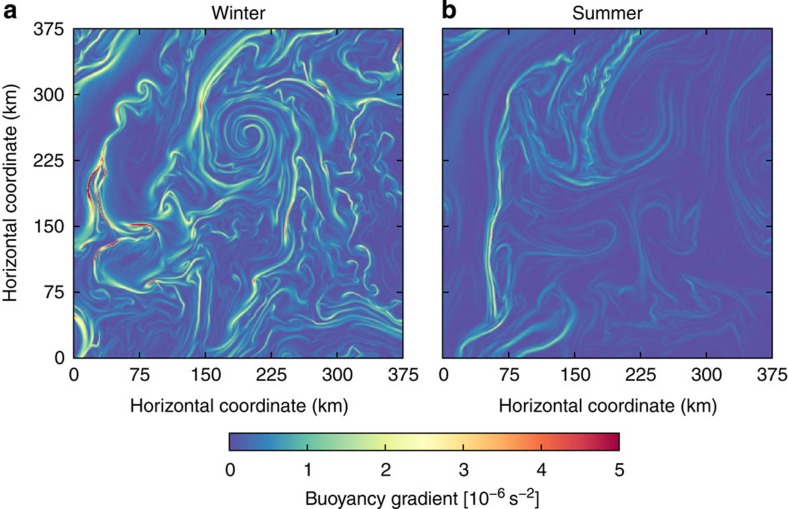
Seasonality in numerical simulations. Surface buoyancy gradient magnitudes from a numerical simulation in the Gulf Stream region in (**a**) winter (February 15) and (**b**) summer (August 15).

These spectra confirm that submesoscale turbulence is more energetic in winter than in summer. The seasonal transition in submesoscale potential energy levels is consistent with the seasonal transition in frontal strength seen in numerical simulations (Fig. 1). In winter, the observed equipartition between kinetic and potential energy, *K(k)/P(k)≃1*, and the *k*^−2^ spectral slopes are likely the result of the turbulent dynamics induced by mixed-layer instabilities and their interaction with the mesoscale straining field, but how these dynamics shape the spectra is currently not understood. The wintertime spectral slopes are also consistent with the mesoscale-driven surface frontogenesis mechanism, but the seasonal transition is not. In summer, the steep energy spectra are consistent with interior quasi-geostrophic turbulence^2^. Eddies generated through baroclinic instability in the thermocline transfer very little energy to submesoscales. The observation that the kinetic energy spectrum is substantially larger than the potential energy spectrum in this range is also consistent with the ratio *K*(*k*)/*P*(*k*)≃4 predicted by interior quasi-geostrophic turbulence^2^,^31^. The emergence of the GM spectrum at scales below 20 km confirms that internal waves dominate these scales and mask an even more dramatic drop-off of geostrophic submesoscale motions in summer, as pointed out in recent literature^31^,^32^.

### Wintertime submesoscale flows

We examine the vertical structure of kinetic energy in the LatMix winter experiment, which provides further evidence for the mixed layer instability mechanism. We use the LatMix data, because the accompanying buoyancy data provide information on the mixed-layer depth. It is roughly constant at 220 m across the transects, which facilitates the interpretation of the vertical structure in observed kinetic energy. The submesoscale kinetic energy is fairly constant throughout the mixed layer at scales larger than 20 km (Fig. 4a). At smaller scales, the energy is slightly enhanced at the surface and at the base of the mixed layer, as expected from Eady dynamics at scales below the deformation radius. The spectra uniformly scale like *k*^−2^ in the mixed layer. Below the base of the mixed layer, the energy decays rapidly; the spectra steepen to *k*^−3^ in the permanent thermocline at scales larger than 20 km (Fig. 4b). At smaller scales, the spectra in the thermocline follow the GM model and internal waves mask a steep drop-off. This vertical structure is consistent with submesoscale turbulence induced by baroclinic instabilities in the mixed layer, but inconsistent with mesoscale-driven surface frontogenesis, for which the vertical decay of submesoscale turbulence would start in the mixed layer^31^.

The observations further suggest that the energetic submesoscale flows in winter are to leading order in geostrophic balance, as required for an inverse energy cascade. Horizontally non-divergent isotropic flows have a distinct signature in the power spectra of transverse and longitudinal velocities: for a flow with a *k*^−2^ kinetic energy spectrum, the ratio *S*^*v*^(*k*)/*S*^*u*^(*k*)≃2 is expected^2^,^31^,^32^,^33^. Error bars are substantial, but a clear trend across scales is evident, which suggests that the observed spectra are roughly consistent with this relation. In the Oleander data, where the large number of transects results in smaller error bars, the relation is roughly satisfied over the entire submesoscale range (Fig. 5a). In the LatMix winter experiment, the relation is roughly satisfied throughout the submesoscale range (Fig. 5b), except for scales smaller than 5 km, where noise may affect the spectra significantly (*cf*. Fig. 3b). This is consistent with a flow that is to leading order geostrophically balanced. Strongly ageostrophic dynamics, on the other hand, have a leading-order horizontal divergence and thus a different ratio. Near-inertial waves forced by strong winter winds or wind- and buoyancy-driven three-dimensional turbulence can thus be ruled out to dominate the submesoscale observations in winter.

Calculation of the Rossby and Froude numbers as functions of scale, Ro(*k*)=[*k*^3^*K*(*k*)]^1/2^/*f* and Fr(*k*)=[*k*^3^*P*(*k*)]^1/2^/*f*, further suggests that the observed wintertime submesoscale flows follow quasi-geostrophic dynamics. Quasi-geostrophic dynamics describe flows with Fr(*k*)∼Ro(*k*)<<1 (for example, see ref. 34). For *k*^−2^ kinetic energy spectra, as observed in winter, Ro(*k*) increases moderately with *k*, approximately like *k*^1/2^. As Ro(*k*) is small in the mesoscale, it remains fairly small throughout the observed submesoscale range (Fig. 5c,d). The condition Fr(*k*)∼Ro(*k*) is satisfied because of the approximate equipartition between kinetic and potential energy (Figs 3b and 5d). Extrapolating Ro(*k*) and Fr(*k*) to smaller scales, we expect that they reach order 1 at order 1 km. The dynamics are thus expected to become strongly ageostrophic—allowing effective transfer of energy to dissipation scales^24^,^25^—at scales smaller than the observed range.

## Discussion

The near-surface observations are characterized by a pronounced seasonal cycle in kinetic and potential energy: submesoscale flows are much more energetic in deep winter mixed layers than in the seasonal thermocline in summer. In contrast, the mesoscale energy remains fairly constant throughout the year. The near-surface submesoscale spectral roll-off changes from a rapid *k*^−3^ in summer to a frontal *k*^−2^ in winter. In winter, submesoscale energies are fairly constant with depth throughout the mixed layer and decay rapidly below its base, transitioning to a *k*^−3^ regime in the main thermocline.

We can rationalize these results in terms of quasi-geostrophic turbulence theory. A very weak forward transfer of energy below the scale of the deep mesoscale instability prevails in the seasonal thermocline in summer and in the main thermocline throughout the year. In winter, deep mixed layers allow a secondary baroclinic instability in the mixed layer that converts potential to kinetic energy in the submesoscale, over a range of scales around the mixed-layer deformation radius between 1 and 10 km. This injection of kinetic energy throughout the depth of the mixed layer drives an inverse cascade of kinetic energy to larger scales and energizes the entire range between the mesoscale and the mixed-layer deformation radius.

The observations are consistent with realistic high-resolution numerical simulations^6^,^7^ and thereby suggest that these simulations capture the leading-order dynamics of submesoscale turbulence. This consistency further builds confidence in the instability mechanisms, because in the simulations one can directly diagnose a seasonal cycle in the release of available potential energy through mixed-layer instabilities and an inverse cascade of kinetic energy thus created^6^,^7^. Although this inverse cascade is not directly diagnosed from data, two crucial ingredients are. First, the lack of a spectral peak at the instability scale indicates that the instability has grown to finite amplitude and become fully turbulent. Second, the diagnosed horizontal non-divergence indicates that the flow is to leading order in geostrophic balance, which is necessary for turbulent scale interactions to preferentially transfer energy to large scales.

The observed seasonal cycle of submesoscale energy levels, the vertical structure of the flow and its horizontal non-divergence in winter are all consistent with the instability mechanism. In conjunction with the results of numerical simulations, the observations therefore strongly favour the hypothesis that the submesoscale is energized through a baroclinic instability in the mixed layer. This consistency, of course, does not constitute a proof of the instability mechanism and further progress can be made by deriving additional falsifiable predictions or by obtaining additional observations. But at the moment, the instability mechanism appears to be the only available explanation for the observations on hand.

Previous studies suggested that mesoscale-driven surface frontogenesis generates most of the submesoscale kinetic energy in the upper ocean^3^. This process appears to be weak in the western subtropical North Atlantic. In summer, when the mixed-layer instability is not active, the submesoscale spectra roll off like *k*^−3^—surface frontogenesis, which can occur year round, would generate steps in the velocity field and thus produce *k*^−2^ spectra. It appears that in this region the summertime flow is dominated by interior quasi-geostrophic turbulence, even close to the surface.

The mixed-layer instability mechanism is likely to dominate the generation of submesoscale kinetic energy in all parts of the ocean that are characterized by deep mixed layers. For example, the mechanism appears to be at work in the entire Gulf Stream and Kuroshio regions in winter, as found in high-resolution numerical simulations^6^,^7^. We also expect this mechanism to be at work in parts of the Southern Ocean, where winter mixed layers can reach as deep as 500 m (ref. 35).

Our results further suggest that a strong seasonal cycle in submesoscale energy is probably characteristic of regions with relatively strong interior potential vorticity gradients reversing sign at depth, such as the western subtropical North Atlantic analysed here^36^. With such potential vorticity profiles, the mesoscale field is generated through a Phillips-type baroclinic instability^37^, which energizes low-mode eddies and transfers little energy to scales smaller than the mesoscale instability scale. Surface buoyancy gradients are relatively weak, rendering any mesoscale-driven surface frontogenesis ineffective. In other regions, where surface buoyancy gradients are larger and deep potential vorticity gradients weaker, Charney-type baroclinic instabilities and mesoscale-driven surface frontogenesis may become a leading-order driver of submesoscale turbulence in the upper ocean^38^.

Submesoscale turbulence is associated with strong flows along steep isopycnals. These generate large vertical fluxes of physical and chemical tracers^4^ and may have an impact on the exchange of these tracers between the mixed layer and the interior ocean. The submesoscale enhancement of fluxes of heat and salt has been suggested to affect the water mass properties and circulation of the permanent thermocline^39^. Furthermore, submesoscale fluxes of nutrients between the nutrient-depleted mixed layer and the nutrient-rich thermocline are believed to play an important role in maintaining primary production in subtropical gyres^40^. It remains to be studied, however, how the seasonality in submesoscale turbulence affects these exchanges. Stommel^41^ argued that the properties of waters subducted from the mixed layer into the permanent thermocline are set in winter, when mixed layers are deepest. Our study has shown that winter is also the time when submesoscale turbulence is most vigorous—just when Stommel's demon opens the trapdoor. An impact of submesoscale turbulence on the physical and chemical properties of interior waters appears plausible.

## Methods

### Numerical simulation

The snapshots of buoyancy gradients shown in Fig. 1 are taken from a numerical simulation of the Gulf Stream region performed with the Regional Ocean Modeling System^42^. This simulation has a horizontal resolution of 750 m and 50 vertical levels. The model domain spans 1,050 km by 900 km, which provides generous padding to the domain shown in the snapshots. Boundary conditions are supplied by a lower-resolution simulation that spans the Atlantic basin^43^. The simulation is forced by daily winds and diurnally modulated surface fluxes. The modelling approach is described in detail in Gula *et al*.^44^.

### *In-situ* observations

**Figure 2:**
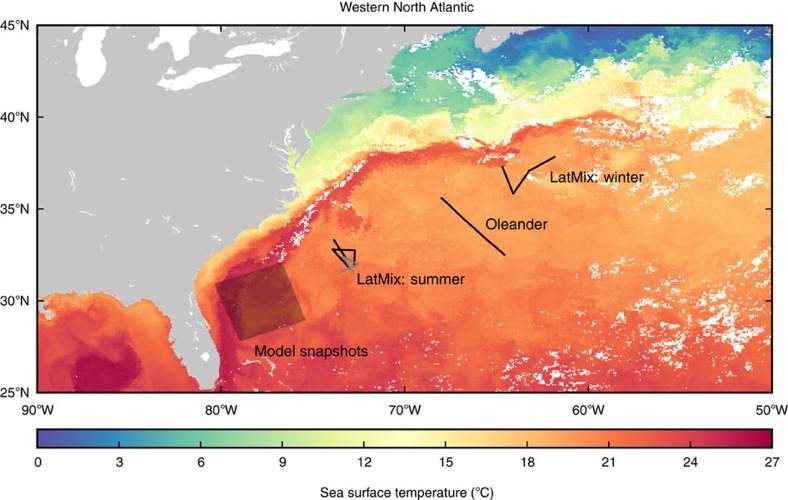
Measurement locations. Locations of velocity transects (black lines), of additional buoyancy transects (dark gray lines) and of the model snapshots shown in Fig. 1 (transparent shading). The colour shading shows sea surface temperatures on 13–20 March 2012 (8-day L3 MODIS Aqua composite of 4 μm nighttime temperature). Missing data are indicated by white shading.

**Figure 3:**
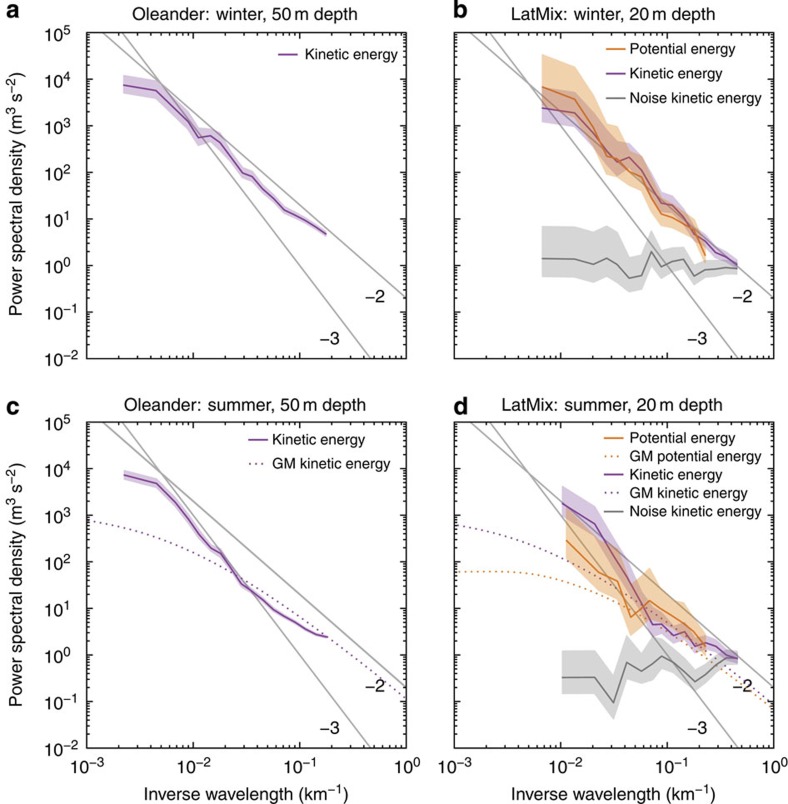
Seasonality in observations. (**a**) Kinetic energy spectrum at 50 m depth for the Oleander winter data. (**b**) Potential and kinetic energy spectra at 20 m depth for the LatMix winter experiment. (**c**) Kinetic energy spectrum at 50 m depth for the Oleander summer data. (**d**) Potential and kinetic energy spectra at 20 m depth for the LatMix summer experiment. The light shadings are 95% confidence intervals. Also shown are the GM model spectra for internal waves in the seasonal thermocline (with parameters from ref. 30), estimates for the noise level of the LatMix velocity data and reference lines with slopes −2 and −3.

**Figure 4:**
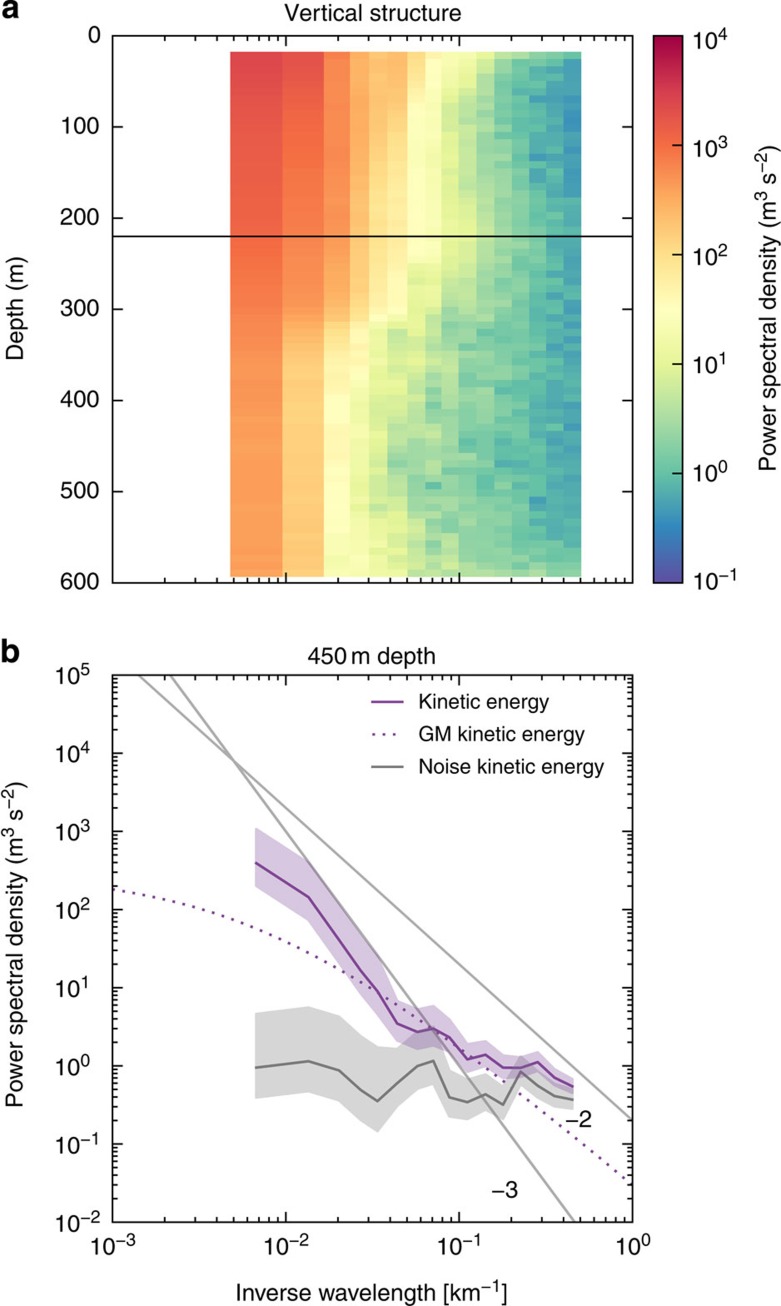
Vertical structure in the LatMix winter experiment. (**a**) Kinetic energy spectrum as a function of depth. The depth of the mixed layer *h*=220 m is indicated by the black horizontal line. (**b**) Kinetic energy spectrum at a depth of 450 m. The light shadings are 95% confidence intervals. Also shown is the GM model spectrum for internal waves in the thermocline (with parameters from ref. 30), an estimate of the noise level of the velocity data and reference lines with slopes −2 and −3.

**Figure 5:**
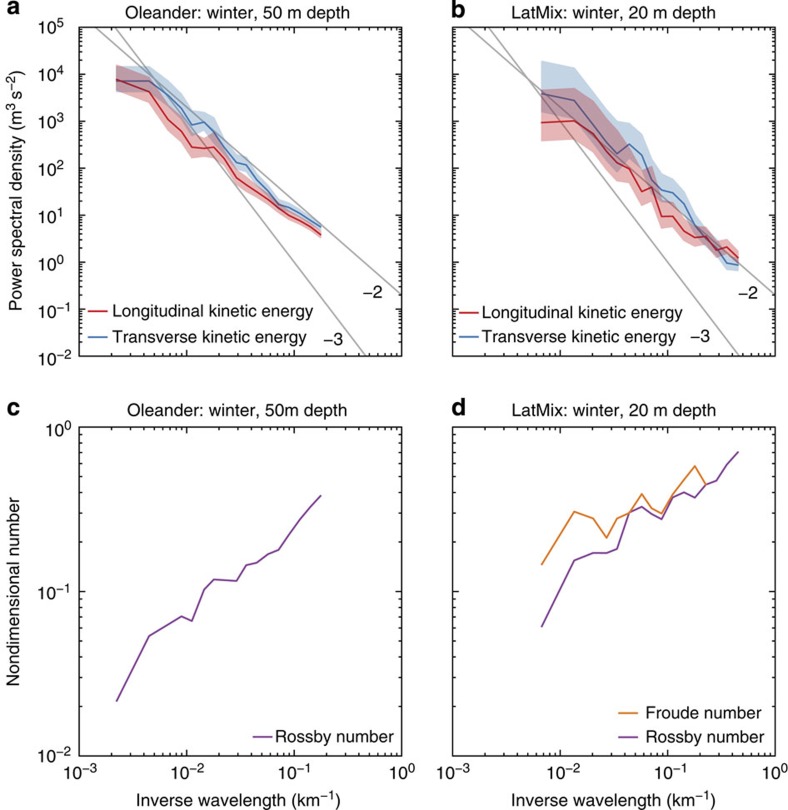
Testing whether the wintertime submesoscale flow is quasi-geostrophic. (**a**) Power spectra of transverse and longitudinal velocities at 50 m depth for the Oleander winter data. (**b**) Power spectra of transverse and longitudinal velocities at 20 m depth for the LatMix winter experiment. (**c**) Rossby number at 50 m depth for the Oleander winter data. (**d**) Rossby and Froude numbers at 20 m depth for the LatMix winter experiment. The light shadings are 95% confidence intervals.

The Oleander data were collected in 2005–2013 with a 75-kHz ADCP, averaged over 8-m depth bins and 4.5- to 6-min intervals, which at a ship speed of about 8 m s^−1^ results in an average spacing of 2.0–2.5 km. Data west of 68°W and north of 36.5°N are discarded; thus, the inhomogeneities of the Gulf Stream extension do not affect the results (Fig. 2). Subsequently, transects with fewer than 190 data points are discarded. The measured velocities are transformed into a coordinate system aligned with the ship track and interpolated with cubic splines onto a regular grid with a spacing of 2.6 km. After selecting for the season, a Hann window is applied and the Fourier transforms are averaged over the different transects to form the spectra. The summer spectrum is an average over 46 transects and the winter spectrum is an average over 11 transects. The spectra are further averaged over ten wavenumber bins per decade. The spectral amplitudes at the smallest resolved scales are affected by the averaging and interpolation procedures, but an assessment of these effects using synthetic signals suggests that the impacts are small and that they do not affect our conclusions, which depend mostly on larger scales.

A comparison with the Oleander data collected with a 150-kHz ADCP in 1994–2004 reveals that although the two data sets are consistent in summer, the 1994–2004 data set exhibits less submesoscale energy in winter than the 2005–2013 data set. More accurate navigational data can be used in the processing of the 2005–2013 data, which makes it more reliable and leads us to suspect that the submesoscale energy is artificially reduced in the 1994–2004 data set during winter.

The LatMix velocity data were collected with 75 kHz ADCPs aboard the *RV Endeavor* (summer) and *RV Atlantis* (winter). They are averaged over 8-m depth bins and 5-min intervals, which at a ship speed of about 3 m s^−1^ results in an average spacing of about 1 km. Four straight large-scale transects are used from the summer experiment and three from the winter experiment (Fig. 2). The winter data were previously analysed by Shcherbina *et al*.^29^. The measured velocities are transformed into a coordinate system aligned with the ship track and interpolated onto a regular grid with a spacing of 1 km. The spectra are obtained following the same procedure as described above for the Oleander data. Estimates of the noise level are calculated as in Shcherbina *et al*.^29^.

The LatMix buoyancy data were collected using Moving Vessel Profilers, sampling the water column 5–100 m in summer and 10–250 m in winter. We use three transects occupied by the *RV Endeavor* in summer (Fig. 2) and three transects occupied by the *RV Atlantis* in winter (same as for velocity). Data from the nearly vertical downcasts are averaged over 1-m depth bins; data from upcasts are discarded. The data are further interpolated onto a regular along-track grid with a spacing of 2 km. The stratification is obtained by fitting a second-order polynomial to the horizontally averaged buoyancy profile in the range 10–100 m in summer and 20–200 m in winter. The spectra are obtained following the same procedure as described above for the velocities.

## Author contributions

J.C. and R.F. planned the research and wrote the manuscript. J.C. analysed the data. J.M. K. planned, collected and processed the Moving Vessel Profiler observations. J.G. performed the model simulations shown in Fig. 1.

## Additional information

**How to cite this article:** Callies, J. *et al*. Seasonality in submesoscale turbulence. *Nat. Commun*. 6:6862 doi: 10.1038/ncomms7862 (2015).

## References

[b1] Stammer D. Global characteristics of ocean variability estimated from regional TOPEX/POSEIDON altimeter measurements. J. Phys. Oceanogr. 27, 1743–1769 (1997).

[b2] Charney J. G. Geostrophic turbulence. J. Atmos. Sci. 28, 1087–1095 (1971).

[b3] Lapeyre G. & Klein P. Dynamics of the upper oceanic layers in terms of surface quasigeostrophy theory. J. Phys. Oceanogr. 36, 165–176 (2006).

[b4] Capet X., McWilliams J. C., Molemaker M. J. & Shchepetkin A. F. Mesoscale to submesoscale transition in the California Current System. Part I: flow structure, eddy flux, and observational tests. J. Phys. Oceanogr. 38, 29–43 (2008).

[b5] Capet X., Campos E. J. & Paiva A. M. Submesoscale activity over the Argentinian shelf. Geophys. Res. Lett. 35, L15605 (2008).

[b6] Mensa J. A. . Seasonality of the submesoscale dynamics in the Gulf Stream region. Ocean Dyn. 63, 923–941 (2013).

[b7] Sasaki H., Klein P., Qiu B. & Sasai Y. Impact of oceanic-scale interactions on the seasonal modulation of ocean dynamics by the atmosphere. Nat. Commun. 5, 5636 (2014). 25501039 10.1038/ncomms6636 PMC4275589

[b8] Stone P. H. Frontogenesis by horizontal wind deformation fields. J. Atmos. Sci. 23, 455–465 (1966).

[b9] Lapeyre G., Klein P. & Hua B. L. Oceanic restratification forced by surface frontogenesis. J. Phys. Oceanogr. 36, 1577–1590 (2006).

[b10] Held I. M., Pierrehumbert R. T., Garner S. T. & Swanson K. L. Surface quasi-geostrophic dynamics. J. Fluid Mech. 282, 1–20 (1995).

[b11] Blumen W. Uniform potential vorticity flow. Part I: theory of wave interactions and two-dimensional turbulence. J. Atmos. Sci. 35, 774–783 (1978).

[b12] Hoskins B. J. & Bretherton F. P. Atmospheric frontogenesis models: mathematical formulation and solution. J. Atmos. Sci. 29, 11–37 (1972).

[b13] Boyd J. P. The energy spectrum of fronts: time evolution of shocks in Burgers' equation. J. Atmos. Sci. 49, 128–139 (1992).

[b14] Haine T. W. N. & Marshall J. Gravitational, symmetric, and baroclinic instability of the ocean mixed layer. J. Phys. Oceanogr. 28, 634–658 (1998).

[b15] Boccaletti G., Ferrari R. & Fox-Kemper B. Mixed layer instabilities and restratification. J. Phys. Oceanogr. 37, 2228–2250 (2007).

[b16] Eady E. T. Long waves and cyclone waves. Tellus 1, 33–52 (1949).

[b17] Blumen W. On short-wave baroclinic instability. J. Atmos. Sci. 36, 1925–1933 (1979).

[b18] Rivest C., Davis C. A. & Farrell B. F. Upper-tropospheric synoptic-scale waves. Part I: maintenance as Eady normal modes. J. Atmos. Sci. 49, 2108–2119 (1992).

[b19] Juckes M. Quasigeostrophic dynamics of the tropopause. J. Atmos. Sci. 51, 2756–2768 (1994).

[b20] Vallis G. K. Atmospheric and Oceanic Fluid Dynamics Cambridge University Press (2006).

[b21] Fjørtoft R. On the changes in the spectral distribution of kinetic energy for two-dimensional, nondivergent flow. Tellus 5, 225–230 (1953).

[b22] Kraichnan R. H. Inertial ranges in two-dimensional turbulence. Phys. Fluids 10, 1417 (1967).

[b23] Marshall J. & Schott F. Open-ocean convection: observations, theory, and models. Rev. Geophys. 37, 1–64 (1999).

[b24] McWilliams J. C., Molemaker M. J. & Yavneh I. From Stirring to Mixing Momentum: Cascades from Balanced Flows to Dissipation in Proc. 12th 'Aha Huliko'a Hawaiian Winter Work 59–66University of Hawaii (2001).

[b25] Capet X., McWilliams J. C., Molemaker M. J. & Shchepetkin A. F. Mesoscale to submesoscale transition in the California Current System. Part III: energy balance and flux. J. Phys. Oceanogr. 38, 2256–2269 (2008).

[b26] Armstrong E. M., Wagner G., Vazquez-Cuervo J. & Chin T. M. Comparisons of regional satellite sea surface temperature gradients derived from MODIS and AVHRR sensors. Int. J. Remote Sens. 33, 6639–6651 (2012).

[b27] Ferrari R. & Rudnick D. L. Thermohaline variability in the upper ocean. J. Geophys. Res. 105, 16857–16883 (2000).

[b28] de Boyer Monteégut C., Madec G., Fischer A. S., Lazar A. & Iudicone D. Mixed layer depth over the global ocean: an examination of profile data and a profile-based climatology. J. Geophys. Res. 109, C12003 (2004).

[b29] Shcherbina A. Y. . Statistics of vertical vorticity, divergence, and strain in a developed submesoscale turbulence field. Geophys. Res. Lett. 40, 4706–4711 (2013).

[b30] Munk W. Internal waves and small-scale processes in Evolution of Physical Oceanography eds Warren B. A., Wunsch C. 264–291The MIT Press (1981).

[b31] Callies J. & Ferrari R. Interpreting energy and tracer spectra of upper-ocean turbulence in the submesoscale range (1–200 km). J. Phys. Oceanogr. 43, 2456–2474 (2013).

[b32] Bühler O., Callies J. & Ferrari R. Wave–vortex decomposition of one-dimensional ship-track data. J. Fluid Mech. 756, 1007–1026 (2014).

[b33] Leith C. E. Atmospheric predictability and two-dimensional turbulence. J. Atmos. Sci. 28, 145–161 (1971).

[b34] Pedlosky J. Geophysical Fluid Dynamics 2nd edn Springer (1987).

[b35] Dong S., Sprintall J., Gille S. T. & Talley L. Southern Ocean mixed-layer depth from Argo float profiles. J. Geophys. Res. 113, C06013 (2008).

[b36] Tulloch R., Marshall J., Hill C. & Smith K. S. Scales, growth rates, and spectral fluxes of baroclinic instability in the ocean. J. Phys. Oceanogr. 41, 1057–1076 (2011).

[b37] Phillips N. A. Energy transformations and meridional circulations associated with simple baroclinic waves in a two-level, quasi-geostrophic model. Tellus 6, 273–286 (1954).

[b38] Roullet G., McWilliams J. C., Capet X. & Molemaker M. J. Properties of steady geostrophic turbulence with isopycnal outcropping. J. Phys. Oceanogr. 42, 18–38 (2012).

[b39] Leévy M. . Modifications of gyre circulation by sub-mesoscale physics. Ocean Model. 34, 1–15 (2010).

[b40] Klein P. & Lapeyre G. The oceanic vertical pump induced by mesoscale and sub mesoscale turbulence. Ann. Rev. Mar. Sci. 1, 351–375 (2009). 10.1146/annurev.marine.010908.163704 21141041

[b41] Stommel H. Determination of water mass properties of water pumped down from the Ekman layer to the geostrophic flow below. Proc. Natl Acad. Sci. USA 76, 3051–3055 (1979). 16592670 10.1073/pnas.76.7.3051 PMC383760

[b42] Shchepetkin A. F. & McWilliams J. C. The Regional Oceanic Modeling System (ROMS): a split-explicit, free-surface, topography-following-coordinate oceanic model. Ocean Model. 9, 347–404 (2005).

[b43] Mason E. . Procedures for offline grid nesting in regional ocean models. Ocean Model. 35, 1–15 (2010).

[b44] Gula J., Molemaker M. J. & McWilliams J. C. Gulf Stream dynamics along the southeastern U.S. seaboard. J. Phys. Oceanogr. 45, 690–715 (2015).

